# The Influence of Mark-Recapture Sampling Effort on Estimates of Rock Lobster Survival

**DOI:** 10.1371/journal.pone.0151683

**Published:** 2016-03-18

**Authors:** Ziya Kordjazi, Stewart Frusher, Colin Buxton, Caleb Gardner, Tomas Bird

**Affiliations:** 1 Institute for Marine and Antarctic Studies (IMAS), University of Tasmania, Hobart, Australia; 2 ARC Center for Excellence in Environmental Decisions, University of Melbourne, Melbourne, Australia; National Taiwan Ocean University, TAIWAN

## Abstract

Five annual capture-mark-recapture surveys on *Jasus edwardsii* were used to evaluate the effect of sample size and fishing effort on the precision of estimated survival probability. Datasets of different numbers of individual lobsters (ranging from 200 to 1,000 lobsters) were created by random subsampling from each annual survey. This process of random subsampling was also used to create 12 datasets of different levels of effort based on three levels of the number of traps (15, 30 and 50 traps per day) and four levels of the number of sampling-days (2, 4, 6 and 7 days). The most parsimonious Cormack-Jolly-Seber (CJS) model for estimating survival probability shifted from a constant model towards sex-dependent models with increasing sample size and effort. A sample of 500 lobsters or 50 traps used on four consecutive sampling-days was required for obtaining precise survival estimations for males and females, separately. Reduced sampling effort of 30 traps over four sampling days was sufficient if a survival estimate for both sexes combined was sufficient for management of the fishery.

## Introduction

Capture-mark-recapture (CMR) modelling is a common method of estimating demographic parameters in field studies where live recapture or resighting is possible [1–3]. To understand the dynamic of animal populations, it is necessary to track the fate of animals for extended periods. CMR studies have been widely used for this purpose in marine ecology and fisheries to investigate abundance [1], survival probability [2] and migration or movement pattern of animals. In addition, fisheries scientists use tag recovery data where tagged animals are fished upon recapture to estimate fishing mortality.

Although CMR methods are widely applied, there is often limited attention given to sampling design. Planning the appropriate intensity and duration of sampling to obtain a specified level of precision is a challenging issue and can bias or affect estimates if there is insufficient data. For example, McKelvey and Pearson [4] reported that 98% of samples collected in small mammal population studies were too small for estimating population density. Similarly, Howe *et al*., [5] found that 15–25 traps could not always provide sufficient data to estimate population density for female black bears.

Precision and accuracy of estimated survival probabilities are known to increase with sample size and duration of sampling [1, 6]. However, sampling intensity and effectiveness may be constrained by environmental and biological factors as well as by the pragmatic constraints of research funding and time. Tagging studies often rely on data collected by trapping and in these studies the sampling efficiency is affected by the catchability of animals, which in turn is affected by environmental and physiological factors. For example, catchability of animals by baited-traps such as lobster [7], crab [8] and crayfish [9] consistently decline in winter. In addition, several biological characteristics of crustaceans including moulting and mating can occur at different times of the year and have been shown to influence the precision and accuracy of survival rate estimates [10, 11].

Perhaps most importantly, research funds and time often place the greatest limitation on CMR studies because this restricts sample size and duration of sampling due to the cost of vessels and labour. Other resourcing constraints include the operational costs of tagging equipment, for example with the cost of transmitters in satellite telemetry [12] or the cost of boat charter, crews and scientists.

It is commonly assumed that CMR methods are robust to restrictions in sampling effort as long as effort remains consistent within the study. However, a number of studies have shown that this assumption may be invalid [6]. For example, sample size and sample duration have been shown to influence estimates of growth rate [13], survival probability [14], population density [5], annual decline in population [15], meta-population estimates of interchange among populations [16], as well as the precision of estimated parameters [12] in both matrix and CMR modelling.

This study examined the effect of sample size and fishing effort required to obtain precise estimates of survival probability using five annual mark-recapture surveys of the southern rock lobster *Jasus edwardsii*.

## Materials and Methods

The research was undertaken in the Crayfish Point Scientific Reserve. Permission to undertake research in this reserve is granted under 14124 (2014–2015), 13108 (2013–2014), 12098(2012–2013), 10062 (2011–2012) from the Tasmanian Department of Primary Industries, Parks, Water and Environment.

No endangered or protected species were affected by this research.

The study was on invertebrates (crustaceans) that do not require animal ethics approval.

### Study area

In CMR studies, precision of survival estimates can be dependent on the size of the population [6] and the size of the sampling area [17, 18]. The effect of varied population size and sampling area was limited in this study by conducting sampling within the Crayfish Point Scientific Reserve (CPSR), Taroona, Tasmania, Australia (42 57’ 08”S 142 21’ 20”E). This reserve contains rocky reef habitat and is surrounded by sand, which limits rock lobster movement [19]. The reserve contains a temperate rocky reef of 1.24 km^2^ with a maximum depth of 15 m. Fishing at the site has been banned since November 1971 and the reserve now holds a dense population of southern rock lobsters [20].

### Field method and simulation

Mark-recapture data were collected for different research projects on *Jasus edwardsii* in five surveys from 2000 to 2005. As individual surveys were originally undertaken to address different projects, there was some variation between surveys in effort including the number of traps used, the number of sampling days and number of lobsters tagged. These factors, in addition to likely changes in catchability and abundance resulted in differences in the number of lobsters tagged and/or recaptured between surveys (Table 1). However, all surveys used consistent sampling protocols and similar equipment. On the first sampling day traps were baited and set in the afternoon. On subsequent days traps were checked in the morning, rebaited and reset. We refer to days when traps are hauled and the contents of the trap sampled as “sampling days”. All untagged lobsters were tagged by a uniquely coded T-bar tag (Hallprint T-bar anchor tag; TBA1, Hallprint Pty Ltd, 27 Jacobson Crescent, Holden Hill, South Australia 5088, Australia) on the ventral side of the first or second abdominal segment. The tag number, length of carapace and sex of each lobster were recorded.

**Table 1:** Summary of sampling data.

	Annual surveys				
	Nov 2000	Dec 01	Jan-Feb 03	Jan-Feb 04	Jan 05
**Sampling-days**	10	7	11	7	10
**Total traps deployed**	891	602	761	403	708
**Traps/day**	89–90	86	69–78	49–63*	68–73
**Lobsters captured**	2362	2085	1714	1022	2032

*The lowest number of traps was 49 which occurred on two days in January-February 2004 survey.

#### Varying sample size

Previous research found that five surveys was the most economical number of surveys for estimating survival probability at a 5% level of precision [21]. The surveys used here were restricted to those that provided an opportunity to subsample up to 1000 tagged lobsters (Table 1). Random subsampling was undertaken with the statistical package R (R core team 2013) from every annual survey, with the number of individuals subsampled set at 200, 250, 300, 400, 500, 750 or 1000. Approximately equal numbers of males and females were selected as the average sex ratio across all surveys was 1.16 (M/F). Recapture history was conducted based on the first survey undertaken in November 2000 and four successive recapture events. This process was repeated ten times by selecting ten independent subsamples for each level of sample size.

#### Varying sampling effort

The effect of sampling effort on estimates of survival rates was examined by subsampling a combination of the number of traps deployed each sampling day and the number of days sampled during each of five annual surveys (Table 1).

To quantify the effect of the number of traps on the precision of lobster survival probabilities, a total of 50, 30 and 15 traps were randomly subsampled from each day of sampling in each survey.

To determine the effect of the number of days of sampling, 7, 6, 4 and 2 sampling days were randomly selected out of the total number of days sampled each survey. As such, 3*4 (= 12) combinations of both the number of traps and days of sampling were applied in each survey, with each of these combinations replicated 10 times. For each subsample, 5-year capture histories were constructed based on lobsters that were tagged in the initial year’s survey (November 2000), and seen or not seen in the four subsequent recapture surveys in the program R (R core team 2013).

Model selection and data analyses were based on Cormack-Jolly-Seber (CJS) models [22–24]. The best model and survival probability of males and females was determined using the capture-recapture analysis in program MARK [25], accessed through R via the RMark package [26].

Survival probability *(Phi)* and recapture probability *(p)* were estimated from each survey data set. The likelihoods of survival probability and recapture probability were estimated incorporating factors of time *(t)* and gender *(g)*. Sixteen parameter combinations of survival and capture probability were estimated with constant survival probability *(Phi(*.*))*, time specific survival probability *(Phi(t))*, gender specific survival probability *(Phi(g))* or time and gender specific survival probability (Phi(g*t)) with constant capture probability *(p(*.*))*, time specific capture probability *(p(t))*, gender specific recapture probability *(p(g))* or time and sex specific recapture probability (p(g*t)). The fully parameterised (saturated) model was Phi(g*t)p(g*t).

Delta AICc (AIC corrected for low sample sizes) was used to determine the most parsimonious model for each analysis from the sixteen candidate set of models [27]. Whilst, the model with the lowest delta AICc value was considered the most parsimonious, all models within 2 units of the lowest delta AICc value (lowest delta AICc value +2) also have substantial support and need to be considered [28].

In this study, the process of data analysis was repeated 10 times by 10 independent subsamples for each level of sample size and effort. To find the best model based on the delta AICc scores among these 10 analyses, each model was coded relative to the lowest Delta AICc score. The codes of 1, 2, 3, 4 and 5 were applied to the models with the value of 0< = lowest Delta AICc<2, 2< = lowest Delta AICc<4, 4< = lowest Delta AICc<7, 7< = lowest Delta AICc<10, and lowest Delta AICc> = 10, respectively. Whilst the model with the lowest mean value among these10 analyses was evaluated as a parsimonious model, all models that were within 0.2 units of the lowest mean value were considered to be likely models.

The best model among the 10 repetitions of each level of subsample was then used to examine the effect of gender and time on survival probability of lobster with varying sample size. Relative standard error (RSE) was used to determine the precision of the estimated survival with low relative standard error, indicating high precision of estimates [12]. Relative standard error was calculated $RSE\;=\;\frac{\bar{SE}}{\bar{X}}$, where $\bar{SE}$ and $\bar{X}$ are standard error and mean of the parameter estimates, respectively.

## Results

### Sample size

The most parsimonious model for estimating survival probability *(Phi)* and recapture probability *(p)* changed from constant (gender and/or time-independent) and/or gender-dependent to only gender-dependent with an increase in the number of tagged lobsters in the sample (Table 2). The number of equally supported models (i.e. within 0.2 units of the most parsimonious model) also decreased from 3 when 250 lobsters were tagged to one when 750 or more lobsters were tagged. Thus the ability to detect gender differences in survival was dependent on the number of lobsters tagged.

**Table 2:** Comparison of model performance with increasing sample size.

Model sample size 250	Coded ΔAICc 250	Model sample size 500	Coded ΔAICc 500	Model sample size 750	Coded ΔAICc 750	Model sample size 1000	Coded ΔAICc 1000
Phi(.)p(g)	1.3*	Phi(g)p(.)	1.5*	Phi(g)p(g)	1.6*	Phi(g)p(g)	2.1*
Phi(.)p(.)	1.4*	Phi(.)p(g)	1.6*	Phi(.)p(t)	2.1	Phi(g)p(g*t)	3.0
Phi(g)p(.)	1.4*	Phi(.)p(.)	1.8	Phi(g)p(t)	2.1	Phi(t)p(.)	3.2
Phi(g)p(g)	2.0	Phi(g)p(g)	1.9	Phi(g)p(.)	2.2	Phi(t)p(g)	3.3
Phi(.)p(t)	2.3	Phi(.)p(t)	2.5	Phi(.)p(.)	2.4	Phi(g*t)p(g)	3.3
Phi(t)p(.)	3.0	Phi(g)p(t)	2.6	Phi(t)p(.)	2.4	Phi(.)p(g)	3.4
Phi(g)p(t)	2.5	Phi(t)p(.)	2.7	Phi(.)p(g)	2.4	Phi(.)p(.)	3.5
Phi(t)p(g)	3.1	Phi(t)p(g)	2.8	Phi(t)p(g)	2.7	Phi(g)p(.)	3.5
Phi(t)p(t)	3.7	Phi(t)p(t)	3.1	Phi(t)p(t)	2.8	Phi(.)p(t)	3.5
Phi(.)p(g*t)	3.4	Phi(g)p(g*t)	3.1	Phi(g)p(g*t)	3.0	Phi(g)p(t)	3.5
Phi(g)p(g*t)	4.1	Phi(g*t)p(.)	3.1	Phi(g*t)p(g)	3.1	Phi(g*t)p(.)	3.6
Phi(t)p(g*t)	4.3	Phi(.)p(g*t)	3.3	Phi(.)p(g*t)	3.3	Phi(.)p(g*t)	3.7
Phi(g*t)p(.)	4.6	Phi(g*t)p(g)	3.6	Phi(g*t)p(.)	3.3	Phi(t)p(t)	4.0
Phi(g*t)p(g)	4.8	Phi(g*t)p(t)	3.9	Phi(t)p(g*t)	3.8	Phi(g*t)p(t)	4.0
Phi(g*t)p(t)	4.6	Phi(t)p(g*t)	4.2	Phi(g*t)p(t)	3.9	Phi(t)p(g*t)	4.1
Phi(g*t)p(g*t)	5.0	Phi(g*t)p(g*t)	4.6	Phi(g*t)p(g*t)	4.3	Phi(g*t)p(g*t)	4.2

(*) indicates models that were considered equally parsimonious (i.e. within 0.2 units of the most parsimonious model)

### Survival probability and precision of survival probability

**Fig 1:**
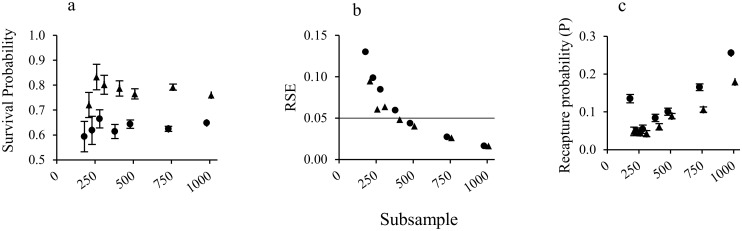
Estimated survival probability (a), RSE of survival probability (b) and recapture (resighting) probability (c) of males (●) and females (▲) at different levels of subsample size (number of individuals).

To determine the survival probability of males and females separated, the most parsimonious model which occurred across all sample sizes (Phi(g)p(g)) was selected. Both estimated survival (Fig 1a) and recapture probability (Fig 1c) increased with sample size. Females had a higher survival probability and lower recapture probability than males. The precision of survival estimates and recapture probability improved with sample size for both sexes as standard errors of estimates decreased (Fig 1b). Although the degree of precision would be determined by managers or assessors of the fishery, in this study a relative standard error (RSE) of 0.05 was chosen to compare the relative precision across different models.

A sample of at least 500 lobsters was required to obtain survival probability estimates with a RSE < 0.05 for both males and females (Fig 1b). Reliable survival estimates for females alone could, however, be obtained from a sample of 400 female lobsters. This resulted in a 5–10% resighting probability of each lobster.

### Sampling effort

As effort, both in number of sampling days and number of traps increased (i.e., from bottom right quadrat in Table 3 to top left quadrat) the survival and recapture probability estimates for the most parsimonious models tended to shift from gender and time independent to gender dependent and time and gender dependent, respectively.

**Table 3:** Dependency of survival probability (Phi) and recapture probability (p) on sex (g), time (t) and/or sex and time (g*t) in the five top models for combinations of sampling days and number of traps. General model is Phi(x)p(y) where “x” and “y” have been shown in the following table as (x)/(y).

Number of traps	Parsimonious models	Number of sampling days							
		7		6		4		2	
**50**	**First**	(g)/(g*t)	1.0*	(.)/(g*t)	1.3*	(.)/(g)	1.8*	(.)/(.)	1.3*
	**Second**	(.)/(g*t)	1.1*	(g)/(g*t)	1.6	(.)/(t)	2.0*	(.)/(t)	1.5*
	**Third**	(t)/(g*t)	2.5	(t)/(g)	1.8	(g)/(g)	2.1	(g)/(.)	1.6
	**Forth**	(g*t)/(g)	3.0	(t)/(g*t)	2.0	(.)/(.)	2.1	(.)/(t)	1.7
	**Fifth**	(g*t)/(g*t)	3.9	(g*t)/(g)	2.8	(t)/(.)	2.1	(g)/(t)	1.7
**30**	**First**	(.)/(g*t)	1.7*	(.)/(g)	2.0*	(.)/(t)	1.7*	(.)/(.)	1.0*
	**Second**	(g)/(g*t)	2.0	(.)/(t)	2.2*	(.)/(.)	1.7*	(.)/(g)	1.4
	**Third**	(g)/(g)	2.6	(g)/(t)	2.3	(.)/(g)	1.8*	(g)/(.)	1.7
	**Forth**	(.)/(g)	2.8	(g)/(g)	2.5	(t)/(.)	1.8*	(g)/(g)	1.9
	**Fifth**	(.)/(t)	2.9	(.)/(.)	2.5	(g)/(.)	2.0	(.)/(t)	2.1
**15**	**First**	(g)/(g)	1.5*	(.)/(g)	1.2*	(.)/(.)	1.0*	(.)/(.)	1.0*
	**Second**	(.)/(g)	1.6*	(g)/(g)	1.4*	(.)/(g)	1.0*	(.)/(g)	1.2*
	**Third**	(.)/(.)	1.8	(.)/(.)	1.6	(g)/(.)	1.3	(g)/(.)	1.3
	**Forth**	(g)/(.)	2.1	(g)/(.)	1.7	(g)/(g)	1.8	(g)/(g)	2.2
	**Fifth**	(.)/(t)	2.7	(t)/(g)	2.8	(.)/(g*t)	2.3	(.)/(t)	3.4

(.) denotes where Phi and/or p in the model were independent of sex and/or time.

(*) indicates the most parsimonious models in the range of x+0.2, where x is the lowest average of the coded ΔAICc value among ten repetitions.

#### Survival probability and precision of survival probability

**Fig 2:**
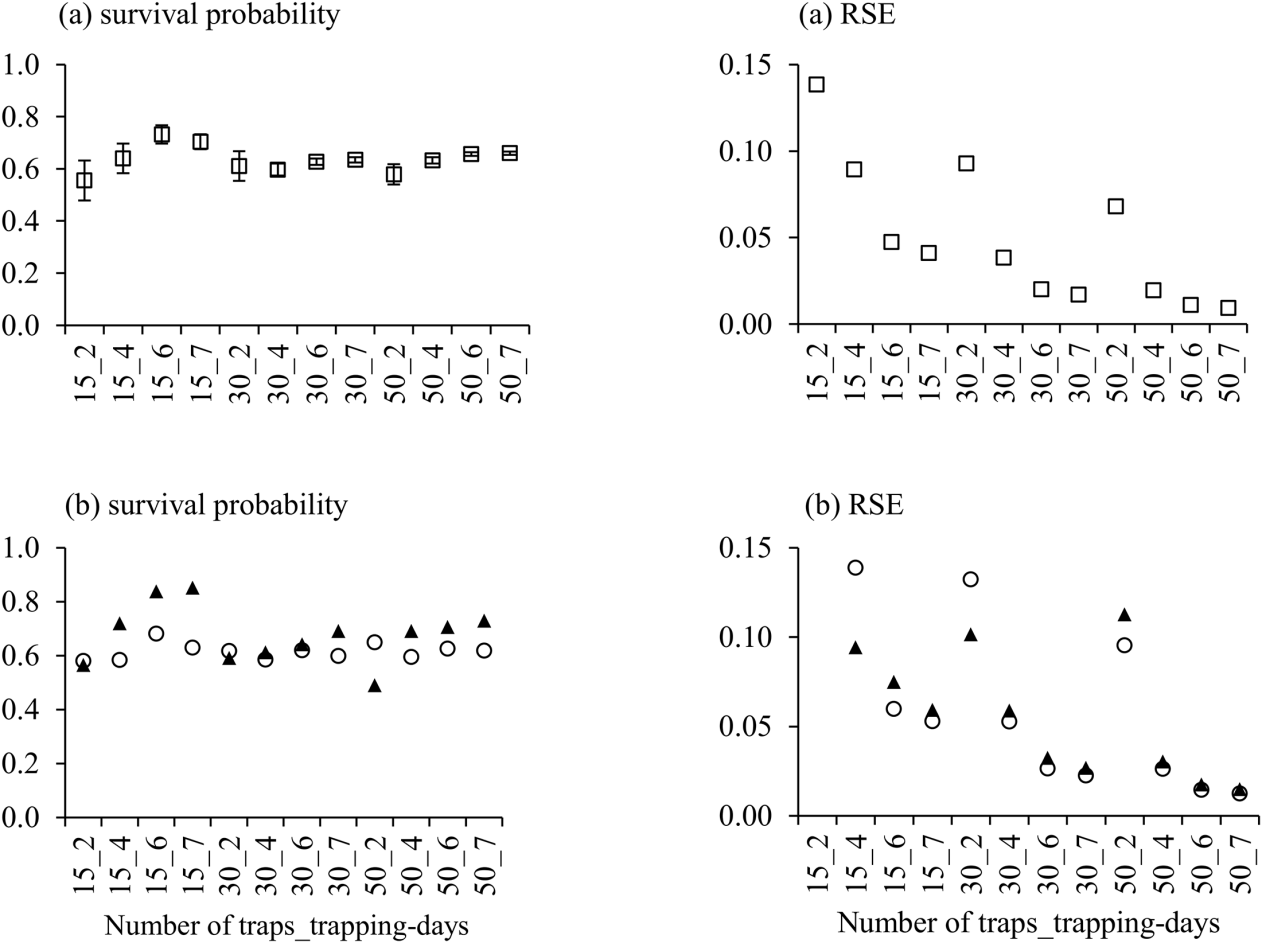
Survival probability *(Phi*) and relative standard error (RSE) of lobsters—combined sexes (a) and different sexes (b) (males (○) and females (▲)), estimated by sex-time independent model *(Phi(*.*)p(g))* and sex dependent model *Phi(g)p(g)* respectively, associated with each combination of fishing effort.

The most common sex-time independent and sex dependent models found across the combinations of the number of traps and sampling days was Phi(.)p(g) and Phi(g)p(g) respectively (Table 3). To determine the appropriate sampling design a precision level (RSE) of 5% was used. If a combined sex model is considered appropriate then 15 traps at 6 days or 30 traps at 4 days would be sufficient. Although 50 traps at 4 days also produced precise estimates, this has not been considered as the required precision, <5% RSE, was not obtained with fewer trap (Fig 2). If the survival probability between the different sexes was considered sufficient to warrant different survival estimates then at least 30 traps for 6 days is required or 50 traps at 4 days (Fig 2). If less precision is required (e.g. 10%), 15 traps for 4 days or 30 traps for 2 days would be the minimum required for estimating annual survival probability for combined sexes and 30 traps for 4 days for separate sexes.

## Discussion

The trade-off between desired precision of survival probability and cost-effectiveness of sampling was evaluated with southern rock lobster data by subsampling from existing data with variation in the number of tagged lobster (sample size) and fishing effort (the number of traps set and number of days sampled). As expected, both the sample size and sampling effort affected the precision and accuracy of survival estimates. Decreasing effort, both in terms of numbers of individuals sampled and the allocation of effort (days sampled and traps set) led to decreased precision in estimates of both survival probabilities *(Phi)* and recapture probabilities *(p)*. Importantly, under-sampling led to biased estimates of survival probability and also minimised the ability to determine differences between sexes. Finally, minimal increase in precision at higher levels of sampling effort, suggests that an optimal ratio of sampling to precision can be achieved which can be used to find a balance in sampling effort that delivers quality data without excessive effort and cost.

Although gender specific *(Phi (g))* was an equally likely model for each of the sample sizes, it was only when 750 or more lobsters were tagged that it had substantially greater support than the combined sexes *(Phi(*.*))* models. However, at the pre-determined RSE of 0.05, 500 tagged lobsters was sufficient to provide precise estimates of survival indicating the importance of both model selections (at 500 tagged lobsters, the gender specific model was not the only parsimonious model) and interrogating the model outputs (i.e. plotting means, standard deviations and RSE).

While the results of this study are specific to lobsters, we suggest that trials similar to the current study, which include variability in sample size and effort, are required to be undertaken before larger tagging programs. In addition to sample size, Conner *et al*., [14] found that season was an important factor with 200 passive integrated transponder (PIT) providing precise survival probability for Steelhead rainbow trout *Oncorhynchus mykiss* in summer, whereas 500 tags could not provide the same precision in autumn or winter.

While catch rates could be used to determine the effort, catch rates can vary considerably and we selected the number of traps set and consecutive sampling days as alternative metrics for determining sampling design.

The number of captured lobsters increased with more sampling effort, that is, with more traps, more trapping days, or both. Not surprisingly, it was possible to obtain a satisfactory estimate of survival by trading off the amount of traps versus days of sampling. Understanding the trade-off between these two aspects of sampling can assist in optimizing sampling strategies [29]. In our particular case, the trade-off involved availability of both staff and vessels.

To reduce the effect of environmental and biological factors on feeding behaviour of lobsters and thus catchability, tagging and recapture events in this study were conducted annually at the same time of year (Table 1), and during periods in which moulting and mating do not occur. Despite these attempts to standardise sampling, it is likely that other processes that could not be controlled by sample design would affect catchability from year to year, including factors such as changes in swell, moon phase [30], food availability and predator abundance. Because of this we speculated that there was potential for different outcomes from short sampling periods with lots of traps each day versus longer sampling periods with fewer traps each day. However, our results suggest that sampling design is free to be driven by the optimal, pragmatic balance between the number of traps and sampling-days that result in the most cost effective and operationally feasible sampling program.

Increasing the number of traps from 15 to 50 enabled more precise survival estimates. Whilst, four days of sampling for 50 subsampled traps was required to obtain precise survival estimates for males and females separately, 30 traps set for four sampling-days was sufficient for obtaining precise survival estimates for combined sexes. Although, fewer traps in a fixed sampling area can impact the effectiveness of bait plumes and reduce catch rates [31], sampling plans based on setting fewer traps over more days may be advantageous in some situations. Whilst more accurate and precise parameter estimates are often pursued in mark recapture studies with the use of more traps [32], data collected with as few as 15 traps over 6 (0.05 RSE) and 4 (0.10 RSE) sampling days did provide parameter estimates for combined sexes and may be suitable depending on the level of precision required by management. This can also be impacted by the logistics of sampling methodology being undertaken as the costs of each additional trap needs to be compared to the cost of labour and vessel usage from adding additional days to the survey.

*A priori* determination of the desired level of precision and accuracy of estimated parameters, population size and sampling area can assist in planning sampling design in a CMR study [6, 33]. This process is necessary for many research projects because of the constraint of research funding which limits the extent of data collection. While this study may provide a guide, we recommend that prior to planning large scale tagging programs, pilot programs be undertaken and analyses such as those undertaken here be used to determine a cost-effective sampling design.
